# Naphthalene exerts substantial nontarget effects on soil nitrogen mineralization processes in a subalpine forest soil: A microcosm study

**DOI:** 10.1371/journal.pone.0217178

**Published:** 2019-05-20

**Authors:** Bo Tan, Fan Yang, Liying Lan, Chengming You, Jian Zhang, Zhenfeng Xu, Yang Liu, Li Zhang, Han Li

**Affiliations:** 1 Institute of Ecology & Forestry, Sichuan Agricultural University, Forestry Ecological Engineering in Upper Reaches of Yangtze River Key Laboratory of Sichuan Province, Alpine Forest Ecosystem Research Station, Soil and Water Conservation and Desertification Control Key Laboratory of Sichuan Province, Chengdu, China; 2 Collaborative Innovation Center of Ecological Security in the Upper Reaches of Yangtze River, Chengdu, China; Friedrich Schiller University, GERMANY

## Abstract

Naphthalene has been widely used to test the functional roles of soil fauna, but its nontarget effects remain uncertain in various soils. To determine whether there is a potential nontarget effect on soil biochemical properties in subalpine forest soil, soils in a subalpine forest on the western Qinghai-Tibet Plateau were treated by naphthalene in microcosms. The responses of soil microbial activity and nutrients to naphthalene were studied following 52 days of incubation. The results showed that the naphthalene application obviously decreased the microbial respiration rate in the first 10 days of the incubation and then increased the rate in the following days of the incubation. Moreover, the naphthalene application did not significantly affect the microbial activities overall, measured as soil microbial phospholipid fatty acid (PLFA) abundances and biomasses, or most enzyme activities (invertase, nitrate reductase and nitrite reductase) during the whole incubation period. However, naphthalene suppressed increases in the DON, NH_4_^+^-N and NO_3_^-^-N contents and urease activity and led to the net mineralization of inorganic N (NH_4_^+^-N + NO_3_^-^-N), in contrast to the net immobilization result in the controls. These results suggest that naphthalene can exert direct nontarget effects on soil microbial respiration and N mineralization processes in subalpine soils. Caution should be taken when using naphthalene to repel soil animals in field experiments.

## Introduction

Soil biota (animals and microbes) are indispensable components and key drivers of soil biogeochemical processes, contributing significantly to organic matter decomposition, nutrient mineralization and greenhouse gas emissions in various ecosystems [1, 2]. While microorganisms are the primary regulators of most of these processes [2, 3], soil animals can play a functional and direct or indirect role in biogeochemical cycling by fragmenting and comminuting litter, regulating microbial activities and altering soil aggregates and structure [4, 5]. Moreover, the composition, diversity and interactions of soil animals and microorganisms modulate carbon and nutrient cycling channels in soil detrital food chains [6, 7]. Consequently, carbon and nutrient cycling cannot be modeled precisely without a full understanding of the functional roles of faunas and their interactions with microorganisms in soil detrital food chains [8, 9].

In recent decades, various methods have been performed to characterize the functional roles of soil animals [10–12]. However, studying the functional roles of soil animals in soil biogeochemical cycling *in situ* without producing nontarget effects on other organisms or affecting the microclimate in surface soil remains challenging [3, 9]. Compared to other biocides (e.g., pyridaben, profenofos and triflumuron), naphthalene (C_10_H_8_) has long been considered to have fewer nontarget effects; this biocide has been used to repel soil fauna and to determine the functional roles of soil fauna in soil and litter organic matter decomposition processes in field experiments [12–14]. Nevertheless, it has long been suspected that naphthalene may indirectly influence these processes through its potential effects on soil microorganisms and nutrients [3, 15]. For example, early microcosm studies suggested that naphthalene might directly affect microbial populations and activities [14]. However, conflicting results showed that naphthalene treatment had weak direct influences on microbial phospholipid fatty acid (PLFA) abundances and carbon dynamics [3]. Moreover, the nontarget effects of naphthalene varied substantially with changes in soil types and incubation conditions [3, 15–17]. Scant research is available regarding the nontarget effects of naphthalene in high-latitude and high-altitude ecosystems, such as alpine and subalpine ecosystems. Consequently, there is a clear need for determining whether there is a direct nontarget effect on the soil biochemical properties in alpine and subalpine forests before conducting field experiments with naphthalene [14, 15].

Here, a microcosm experiment was conducted by adding naphthalene to subalpine forest soil in Southwest China, and the nontarget effects of such additions on soil microbial activities (respiration, PLFA abundance, microbial biomass and enzyme activity) and soil nutrients (dissolved organic matter and inorganic N) were determined. The aims were (1) to assess whether there was a potential nontarget effect on the soil biochemical properties in the subalpine forest soil and (2) to test the hypotheses that naphthalene application might have a stronger nontarget effect on the soil biochemical properties of nitrogen cycling processes than on those of carbon cycling processes because previous studies have suggested naphthalene has a stronger influence on soil inorganic nitrogen availability [14, 16].

## Materials and methods

### Ethics statement

We received permission from the Lixian Forestry Bureau to collect the tested soil in a local forest in 2015. In this study, only a limited number of soil samples were collected to conduct a microcosm experiment, and thus, our work had negligible influences on the function of the broader ecosystem. In addition, this study was carried out in compliance with the laws of the People's Republic of China. The research did not involve measurements of humans or animals, and no endangered or protected plant species were involved.

### Experimental design

In October 2015, approximately 20 kg of tested soil was collected in a secondary fir forest at the Long-term Research Station of Alpine Forest Ecosystems (31°18′N, 102°56′E, 3023 m *a*.*s*.*l*), Southwest China. Soil was collected from five plots (2 m × 2 m size) in the forest using a soil auger (15 cm depth and 5 cm diameter) and mixed after removing visible debris and fresh litter. The soil type was a Cambic Umbrisol according to the IUSS Working Group WRB [18], and the basic soil chemical properties (0–15 cm depth) were as follows: pH 6.5 ± 0.3, soil bulk density 1.04 ± 0.11 g kg^-1^, total organic carbon 153.9 ± 27.4 g kg^-1^, total nitrogen 7.8 ± 1.3 g kg^-1^ and phosphorus 0.9 ± 0.1 g kg^-1^ [19]. The collected soil was sieved (2 mm) and then mixed. Stones, visible animal and plant residues and live macrofuana were removed (e.g. earthworms and millipedes), and the samples were air-dried for the soil microcosm experiment.

Nighty replicate microcosms were constructed with 450 ml clear glass culture bottles (17.5 cm high x 16 cm i.d.). Eighty of the microcosms were filled with 50 g of air-dried soil, and the remaining ten empty microcosms served as blanks. Microarthropods and nematodes in each soil microcosm were eliminated according to the description of Blair et al. [14]. In brief, the soil microcosms were microwaved in a 700-W microwave oven for 120 s and capped for 12 h. This microwave treatment was repeated three times. The soil moisture (w/w) in the microcosms was adjusted to 45% with a suspension of soil microorganisms. The suspension was prepared by homogenizing 200 g of sampled soil in 1.5 liters of deionized water and filtering the homogenate through a 5 μm nucleopore filter [14].

### Soil incubation and respiration measurement

The microcosms consisted of a control group and a treatment group, with 45 microcosms (40 soil microcosms and 5 blanks) for each group. The treatment group then received 0.35 g of naphthalene per bottle at the beginning of the experiment. The other group served as a control (without naphthalene). The soil incubation lasted for 52 days, and two additional naphthalene applications (0.35 g) were performed on days 17 and 38. The total application rate is consistent with field application rates (100 g m^-2^) [14].

The soil microbial respiration rate was allowed to stabilize for three weeks. Before the incubation, plastic vials (8 cm high x 10 cm i.d.) containing 20 ml of 0.01 N NaOH were placed into culture bottles. Following the incubation, the culture bottles were capped and store at 10°C and 45% moisture in temperature-controlled biochemical incubators. The culture temperature and moisture were consistent with our previous field monitoring results [19]. The soil microbial respiration rate in each culture bottle was estimated by determining the carbon dioxide (CO_2_) production following the sampling schedule for 52 days. CO_2_ production was determined by titration with 0.02 N HC1 following the addition of 1 ml of 1 N BaCl [20]. Empty bottles without soil were used as controls.

### Soil sampling and chemical analysis

Soil sampling was performed on days 3, 10, 17, 24, 31, 38, 45 and 52 following the incubation. At each sampling time, 10 soil microcosms (5 controls and 5 naphthalene treatments) were sampled after soil respiration measurements were terminated. Fresh soils were kept in refrigerators at 4 ºC and -70 ºC for soil chemical and microbial analysis, respectively.

The soil extractable N (NH_4_^+^-N and NO_3_^-^-N) was extracted with 2 M KCl and then measured by the method of Lu [21]. The soil dissolved organic carbon (DOC) and dissolved total nitrogen (DTN) were extracted with 0.5 M K_2_SO_4_ [22, 23]. DOC and DTN in the extracts were quantified by a TOC–VcPH+TNM–1 C/N analyzer (Shimazu Inc., Kyoto, Japan). The soil dissolved organic nitrogen (DON) was calculated as follows: DON = DTN − NH_4_^+^-N − NO_3_^-^-N [23]. The soil microbial biomass carbon (MBC) and nitrogen (MBN) were determined by the chloroform fumigation extraction method with a conversion factor of 0.45 for MBC and 0.54 for MBN [24, 25]. The soil enzyme activities of invertase (mg glucose g^−1^ soil dry weight (DW) d^−1^) and urease (mg NH_4_^+^-N g^−1^ soil DW d^−1^) were measured according to the methods of Wang et al. [26] and Lu [21], respectively. Soil nitrate reductase and nitrite reductase activities (mg NO_2_^-^-N g^-1^ soil DW d^-1^) were analyzed according to Xiong et al. [27].

The soil microbial PLFAs were extracted and quantified according to the methods of White et al. [28] and He et al. [29] with partial modifications. The sum of all subsequently described PLFAs was used as a proxy for the total microbial biomass. We used the sum of i15:0, a15:0, i16:0, i17:0 and a17:0 as gram-positive bacterial markers [30, 31], 16:1ω7c, 16:1ω9c, cy17:0, 18:1ω7c, and cy19:0 as gram-negative bacterial markers [32] and 15:0, 16:0, 16:1ω5t, 17:0, 18:00 and 20:5 as general bacterial markers [33, 34]. The gram-positive, gram-negative and general bacterial markers were summed to give the total bacteria. We used the sum of 18:3, 18:1ω9c, 18:2ω6, 9c and 20:1ω9c as fungal markers to represent the total fungi [35, 36]. The detailed gas chromatography–mass spectrometry (GC-MS) conditions were described by Liu et al. [9].

### Data calculation and statistical analyses

The net ammonification, nitrification and inorganic N mineralization at the end of the incubation (52 days) was calculated as the differences in the NH_4_^+^-N (ammonium), nitrate (NO_3_^-^-N) and inorganic N (NH_4_^+^-N + NO_3_^-^-N) contents in the microcosms between the start and end of the incubation. Moreover, data for quantifying the effects of naphthalene addition on soil biochemical properties were calculated as the differences in the average values of the measured variables between the naphthalene treatments and the controls during the whole incubation period.

For specific sampling times, Student’s independent-sample t-test was used to compare the effects of naphthalene application. We used repeated measures of analysis of variance (ANOVA) to test the effects of naphthalene application, sampling time, and their interactions on the measured variables. Differences were considered significant at *P* < 0.05 level for all analyses. All statistical analyses were performed using SPSS 18.0 software package for Windows (SPSS Inc., IL, USA).

## Results

### Naphthalene effects on the soil microbial respiration rate

**Fig 1:**
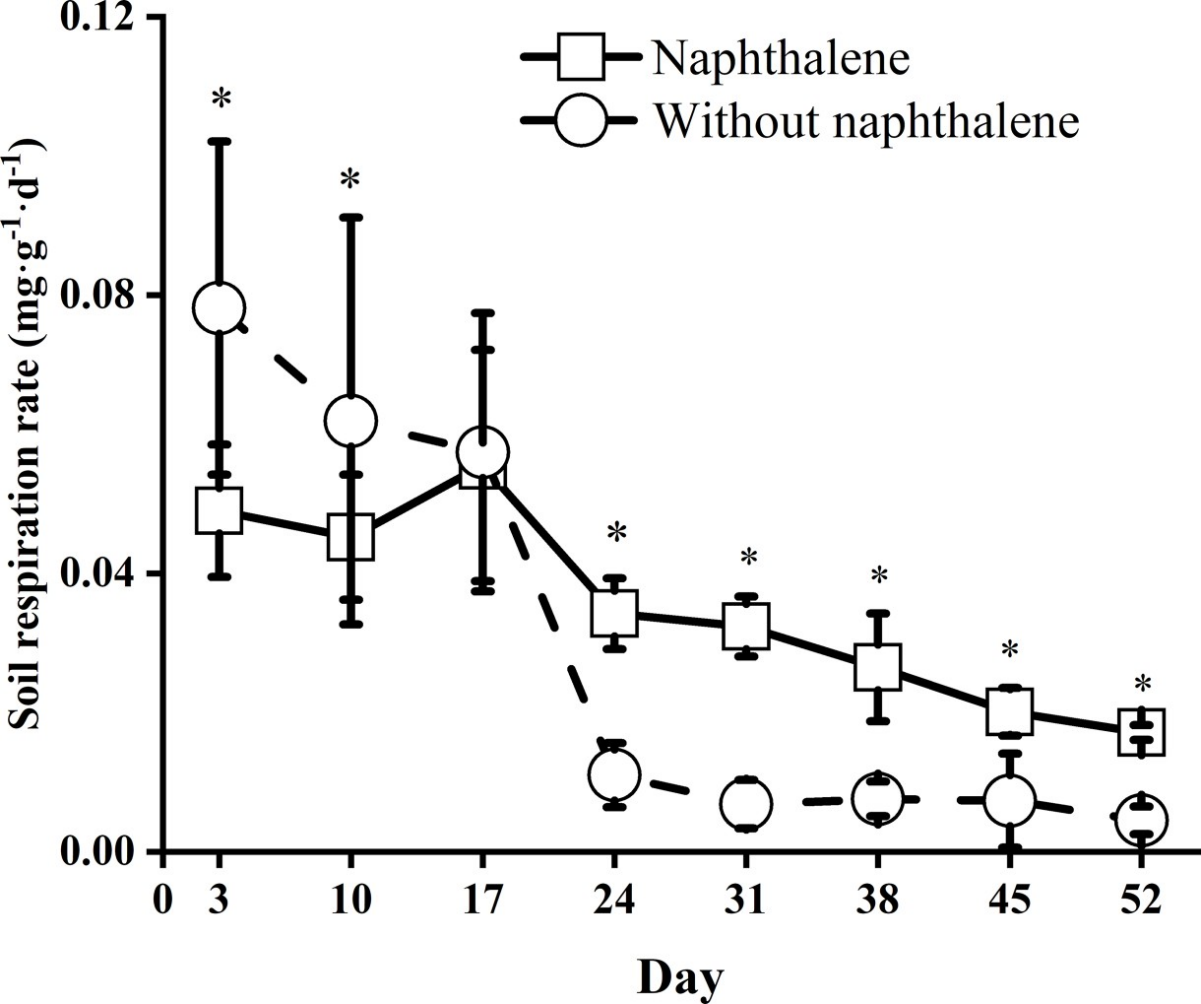
Soil microbial respiration rates in subalpine forest soil of microcosms treated with and without naphthalene. Asterisks indicate significant (*P<0.05) differences between the treatments with naphthalene and without naphthalene at the same sampling time.

The soil microbial respiration rates in both the naphthalene and control (without naphthalene) microcosms varied significantly (*F* = 303.20, *P* = 0.001) over time (Table 1). Tests for time dynamics showed a decreasing trend throughout the experiment (Fig 1). Compared to the control, the naphthalene application obviously (*F* = 30.45, *P* = 0.001) decreased the soil microbial respiration rates in the first 10 days following the incubation and then increased the rates to a higher level between days 24 and 52 of the experiment.

**Table 1:** Repeated measures ANOVA results of the responses of the soil respiration rate, microbial phospholipid fatty acid (PLFA) abundance, microbial biomass, nutrient content and enzyme activities to naphthalene application and sampling time.

Variables	Naphthalene (N)			Time (T)			N × T		
	*df*	*F*	*P*	*df*	*F*	*P*	*df*	*F*	*P*
Soil respiration rate	1	30.45	<0.001**	7	303.20	<0.001**	7	62.86	<0.001**
Bacterial PLFAs	1	4.58	0.099	7	20.68	<0.001**	7	4.59	0.002**
Fungal PLFAs	1	0.36	0.593	7	9.38	<0.001**	7	5.92	0.001**
Fungal/bacterial PLFAs	1	0.36	0.570	7	2.35	0.040*	7	2.78	0.018*
G^+^ PLFAs	1	6.65	0.061	7	56.3	<0.001**	7	3.57	0.007
G^-^ PLFAs	1	7.58	0.051	7	4.82	0.001**	7	3.45	0.009**
G^+^/G^-^ PLFAs	1	10.85	0.017*	7	8.87	<0.001**	7	2.99	0.012*
MBC	1	0.06	0.818	7	5.16	<0.001**	7	0.36	0.940
MBN	1	1.03	0.339	7	6.07	<0.001**	7	0.12	0.999
MBC/MBN	1	0.16	0.707	7	12.42	<0.001**	7	2.44	0.035*
Dissolved organic carbon	1	0.06	0.813	7	101.87	<0.001**	7	74.45	<0.001**
Dissolved organic nitrogen	1	68.46	<0.001**	7	7.21	<0.001**	7	6.59	<0.001**
NH_4_^+^-N	1	445.28	<0.001**	7	26.62	<0.001**	7	56.80	<0.001**
NO_3_^-^-N	1	62.06	<0.001**	7	11.37	<0.001**	7	18.97	<0.001**
Invertase	1	2.11	0.185	7	7.98	<0.001**	7	1.12	0.230
Urease	1	22.28	0.002**	7	240.05	<0.001**	7	5.63	0.002**
Nitrate reductase	1	0.30	0.601	7	35.90	<0.001**	7	1.02	0.428
Nitrite reductase	1	1.63	0.238	7	26.30	<0.001**	7	0.75	0.355

N, naphthalene treatment; T, sampling time. G^+^, gram-positive bacteria; G^-^, gram-negative bacteria; MBC, microbial biomass carbon; and MBN, microbial biomass nitrogen. Asterisks indicate significant (*P<0.05, ** *P*<0.01) differences between the control and the naphthalene treatments over the whole of the experiment.

### Naphthalene effects on the soil microbial PLFAs

**Fig 2:**
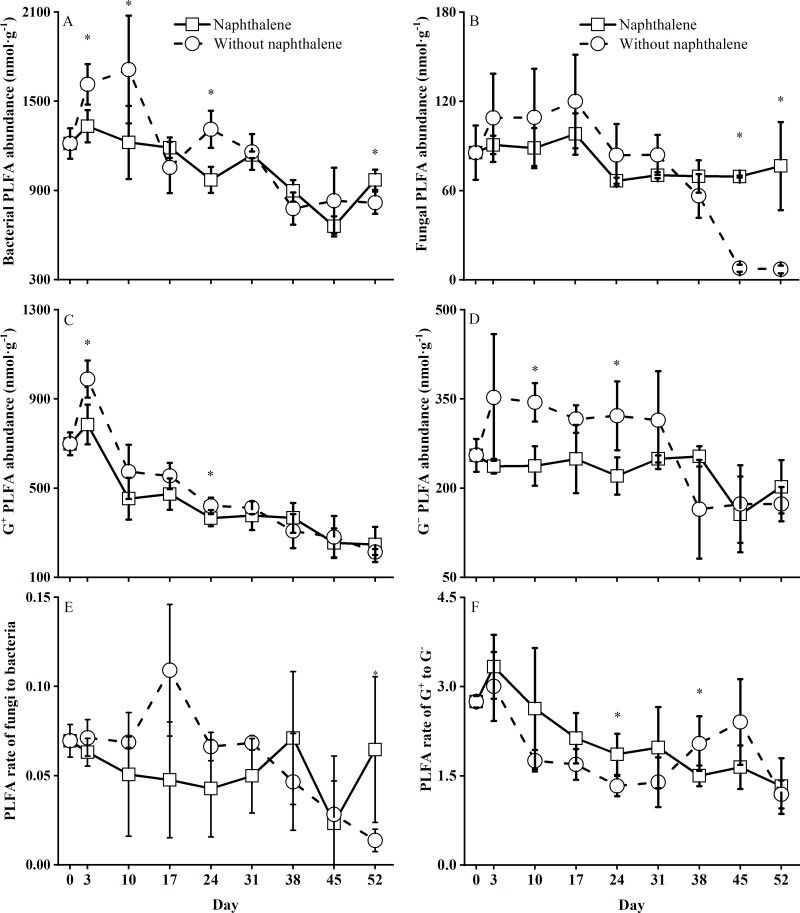
Soil microbial phospholipid fatty acid (PLFA) abundances in subalpine forest soil microcosms treated with and without naphthalene. The values represent the means ± the standard error (SE) (n = 5). Asterisks indicate significant (*P<0.05) differences between the treatments with naphthalene and without naphthalene at the same sampling time.

The abundances of PLFAs (bacteria, fungi, gram-positive (G^+^) bacteria and gram-negative (G^-^) bacteria) were not significantly (all *P* > 0.05) affected by the naphthalene application, but the interaction effect of naphthalene application and sampling time was significant (all *P* < 0.05) on these PLFA abundances (Table 1). Compared to the control, the naphthalene application decreased the abundance of PLFAs and the ratio of fungal PLFAs to bacterial PLFAs before the third naphthalene application on day 38 (Fig 2A–2E). Then, the abundance and ratio of fungal PLFAs and bacterial PLFAs significantly increased until the end of the incubation (Fig 2A, 2B and 2E). Conversely, the ratio of G^+^ to G^-^ bacteria in the naphthalene microcosms increased before the third naphthalene application on day 38 but did not significantly differ in the following incubation (Fig 2F).

### Naphthalene effects on the soil microbial biomass

**Fig 3:**
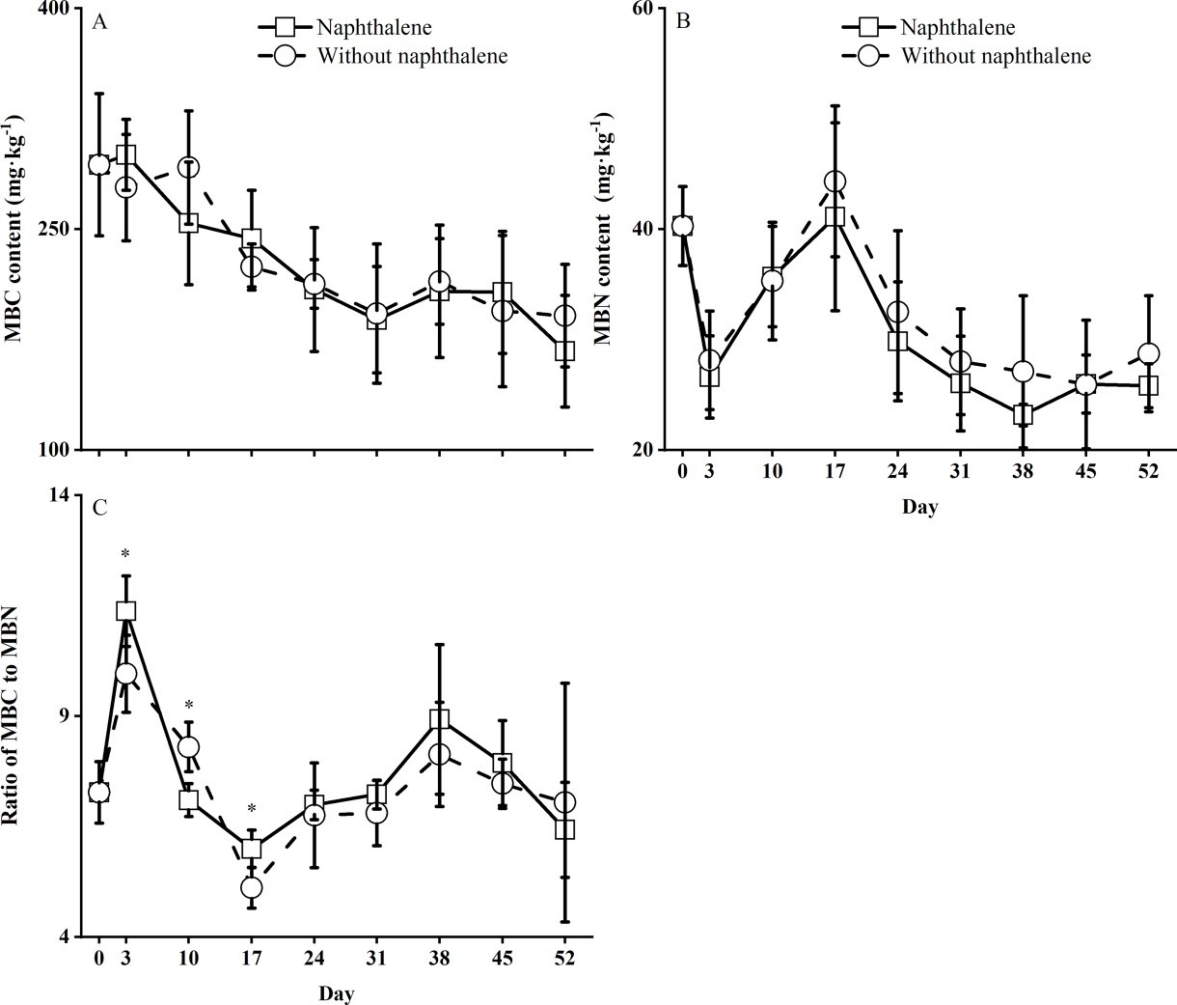
Soil microbial biomass contents in subalpine forest soil microcosms treated with and without naphthalene. The values represent the means ± the standard error (SE) (n = 5). Asterisks indicate significant (*P<0.05) differences between the treatments with naphthalene and without naphthalene at the same sampling time.

Sampling time had a significant (*P* < 0.001) influence on the soil microbial biomass in the microcosms (Table 1). Tests for time trends showed a similar dynamic for the soil MBC and MBN and the ratio of MBC to MBN in both the naphthalene and control microcosms (Fig 3). There was a nonsignificant effect of naphthalene on the contents of MBC (*F* = 0.06, *P* = 0.818), MBN (*F* = 1.03, *P* = 0.339) and the ratio of MBC to MBN (*F* = 0.16, *P* = 0.707) during the whole incubation period, but the interaction of naphthalene application and sampling time exerted a significant (*F* = 2.44, *P* = 0.035) influence on the ratio of MBC to MBN (Table 1).

### Naphthalene effects on the soil dissolved organic matter and inorganic N

**Fig 4:**
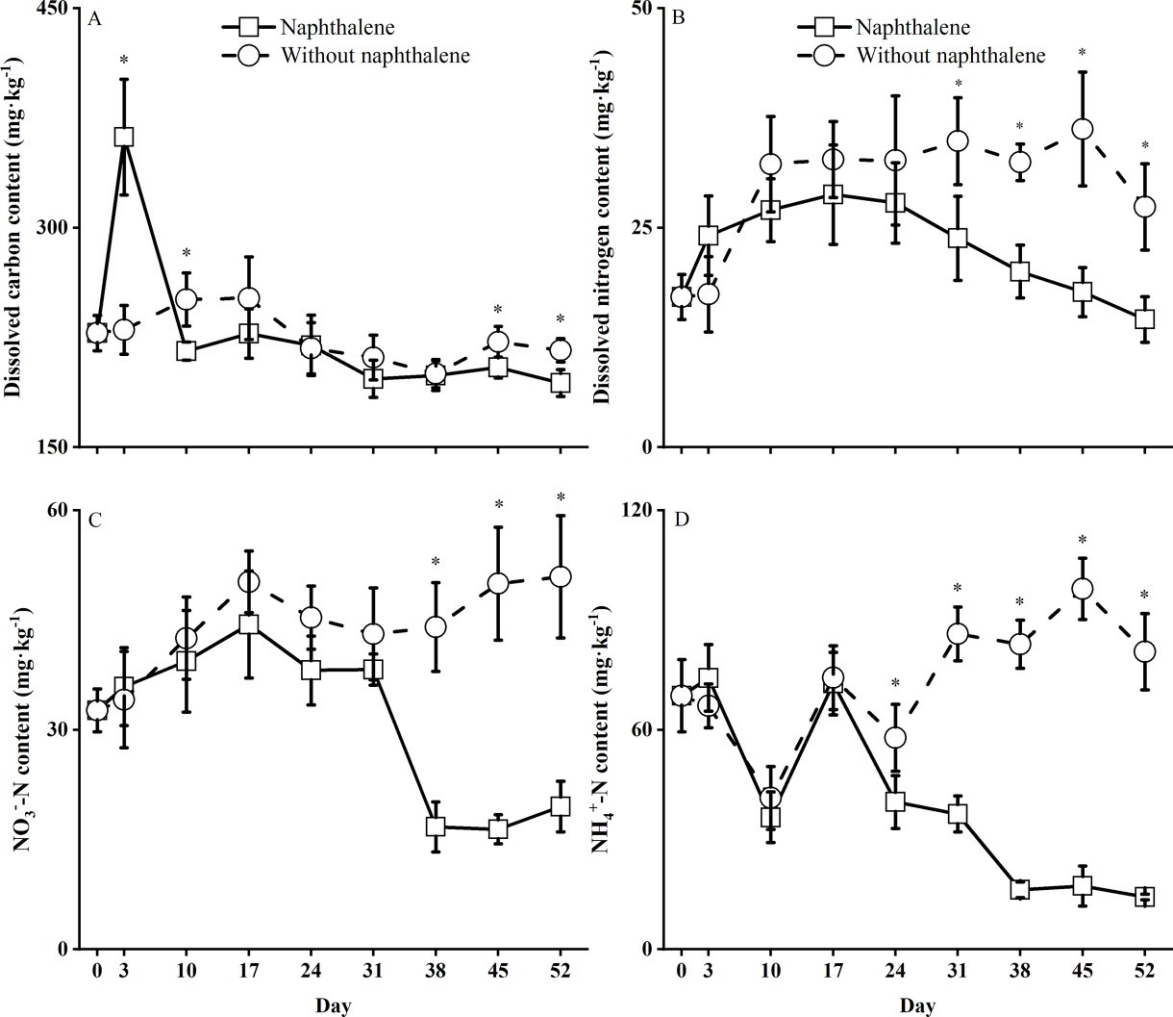
Soil dissolved organic matter and extractable N contents in subalpine forest soil microcosms treated with and without naphthalene. The values represent the means ±the standard error (SE) (n = 5). Asterisks indicate significant (*P<0.05) differences between the treatments with naphthalene and without naphthalene at the same sampling time.

The DOC, DON and inorganic N (NH_4_^+^-N and NO_3_^-^-N) contents in both the naphthalene and control microcosms changed significantly (*P* < 0.001) over time during the experiment. However, the DOC content was not significantly (*F* = 0.06, *P* = 0.813) affected by the naphthalene application (Table 1, Fig 4). Compared to the control, the naphthalene application triggered a sharp increase in DOC content in the first 3 days following the incubation and then decreased the content to a lower value that was maintained for most of the study (Fig 4A). Moreover, the contents of DON and inorganic N were not significantly (*P* > 0.05) influenced by the naphthalene application in the first 17 days of the incubation but started to rapidly decline following the third naphthalene application on day 38 (Fig 4B–4D). In addition, the final contents of soil inorganic N were significantly different between the treatments and controls (Table 2). The absolute contents of soil inorganic N increased by approximately 30% in the controls, while the absolute contents of soil organic N in the treatments decreased by approximately 68% (Table 2).

**Table 2:** Soil net ammonification (NH_4_^+^-N), nitrification (NO_3_^-^-N) and inorganic N (NH_4_^+^-N + NO_3_^-^N) mineralization over the course of the experiment in subalpine forest soil microcosms treated with naphthalene and without naphthalene.

Variables	Without naphthalene			Naphthalene		
	Initial (mg·kg^-1^)	End (mg·kg^-1^)	NM (mg·kg^-1^)	Initial (mg·kg^-1^)	End (mg·kg^-1^)	NM (mg·kg^-1^)
NH_4_^+^-N	69.32±9.86	81.32±10.47a	12.00±12.28a	69.32±9.86	14.30±0.76b	-55.03±7.67b
NO_3_^-^-N	32.65±2.92	50.91±8.34a	18.25±7.40a	32.65±2.92	19.50±3.45b	-13.15±3.22b
Inorganic N	101.98±9.04	132.23±13.32a	30.25±12.78a	101.98±9.04	33.80±2.96a	-68.18±8.75b

NM, net mineralization rate. The nitrogen content is based on the analysis of five individual microcosms from each treatment before and after 52 days. The values represent the means ± the standard error SE (n = 5). Letters indicate significant differences (*P*<0.05) based on Student’s t-test

### Naphthalene effects on the soil enzyme activity

**Fig 5:**
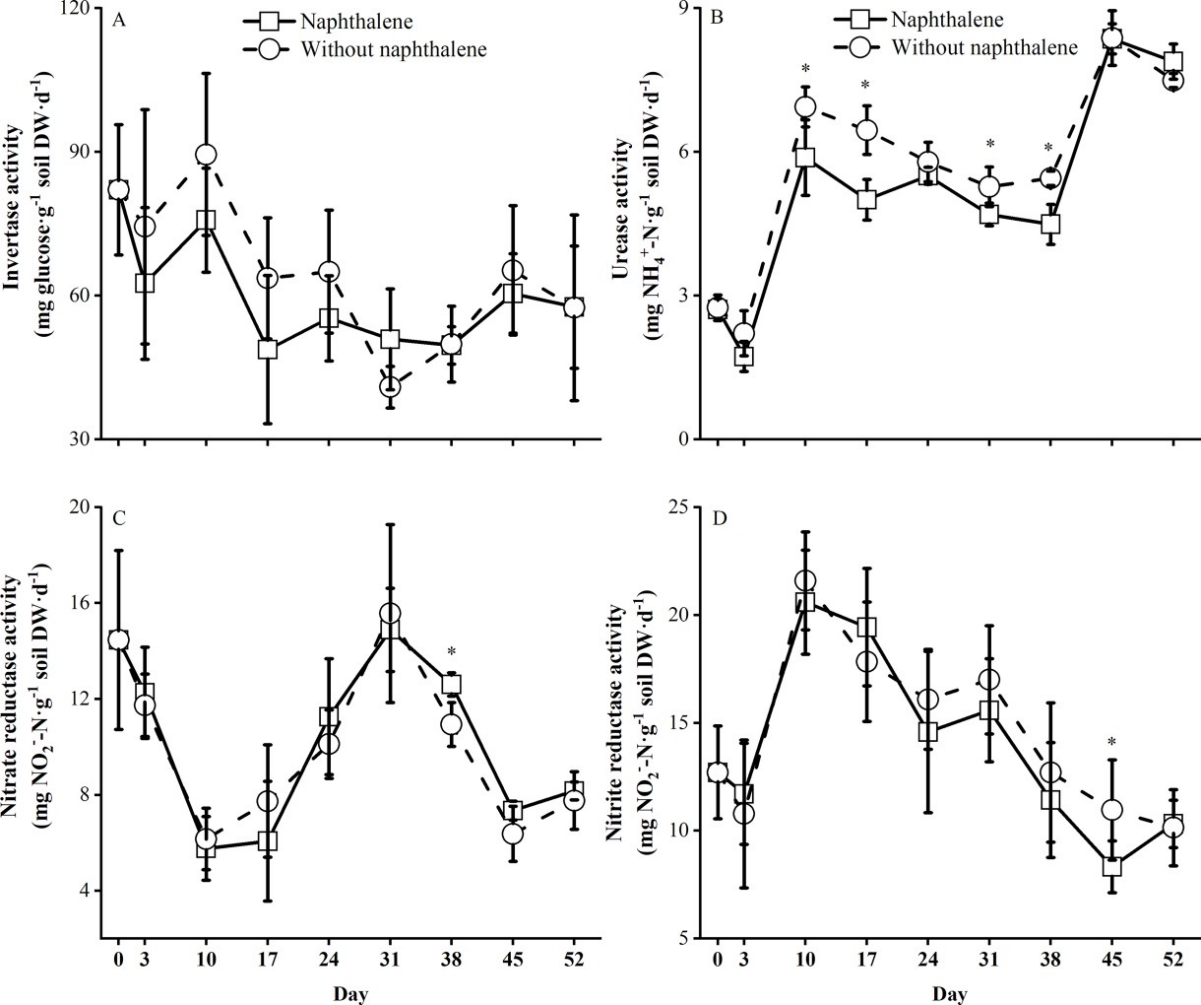
Soil enzyme activities in subalpine forest soil microcosms treated with and without naphthalene. The values represent the means ±the standard error (SE) (n = 5). Asterisks indicate significant (*P<0.05) differences between the treatments with naphthalene and without naphthalene at the same sampling time.

The soil enzyme activity was significantly affected (*P* < 0.001) by the sampling time (Table 1), and the dynamics of these changes were similar to those found in the microcosms (Fig 5). The activities of invertase, nitrate reductase and nitrite reductase in both the treatments and controls were not significantly different (Table 1). Compared to the control, the naphthalene application decreased urease activity to a significantly lower level (*F* = 22.48, *P* = 0.002) during most of the experiment (Fig 5B), and the interaction effect of naphthalene application and sampling time exerted a significant (*F* = 5.63, *P* = 0.002) influence on urease activity (Table 1).

## Discussion

**Fig 6:**
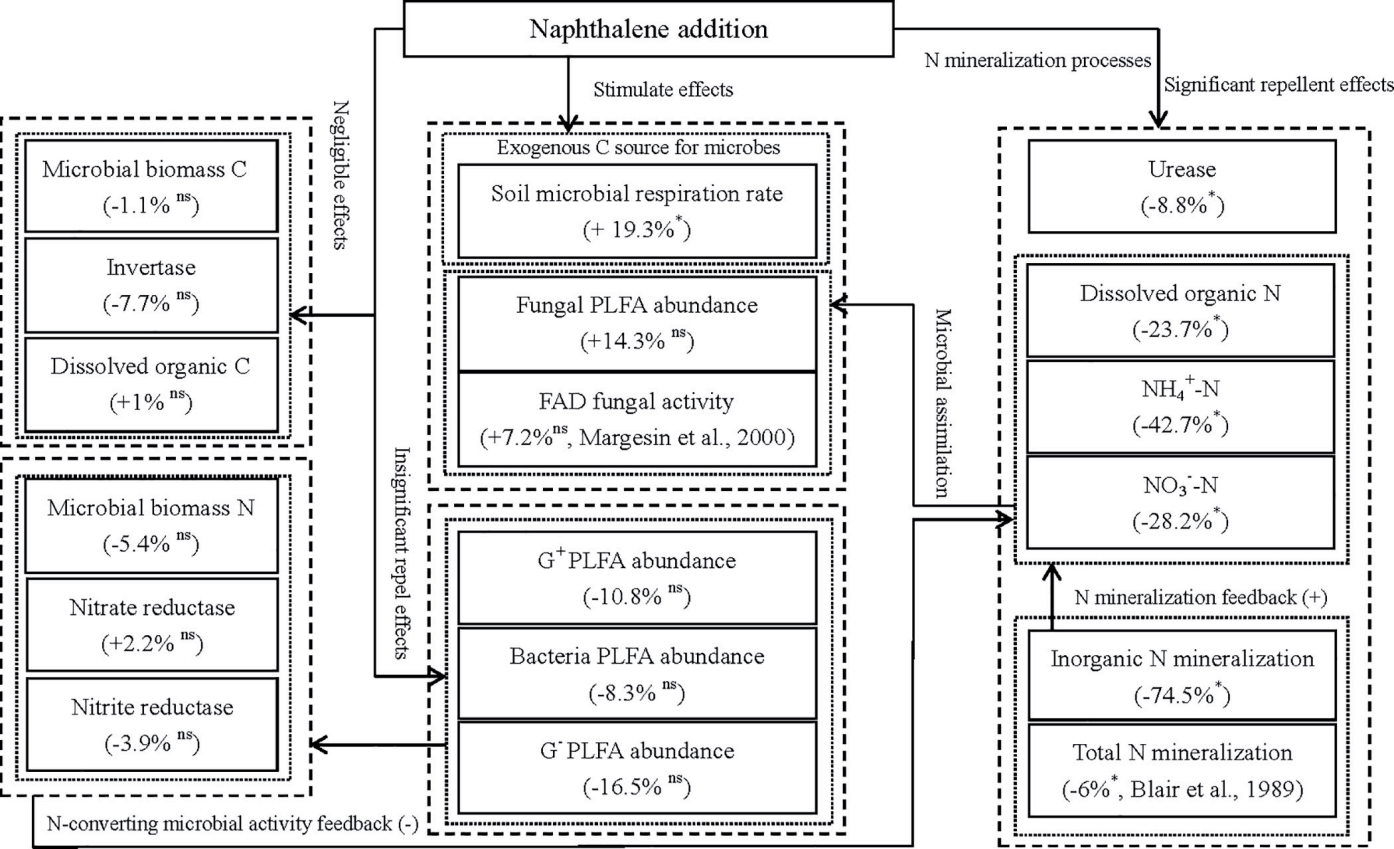
A quantitative framework for the nontarget effects of naphthalene addition on soil biochemical properties. +, positive;–, negative; *, *P* < 0.05; ns, nonsignificant; ^#^ data for FAD fungal activity and total N mineralization are from Margesin et al. (2000) and Blair et al. (1989), respectively.

In recent decades, naphthalene has been widely used as a biocide to reduce or eliminate target groups of soil fauna in field experiments in the study of ecological functions of soil fauna [3, 10, 12, 17]. However, whether naphthalene has potential nontarget effects on soil microorganisms and nutrients has long been debated [3, 14, 15]. Previous soil and soil-litter microcosm studies have suggested that naphthalene might stimulate microbial respiration [14, 37], microbial biomass [15, 38], abundance and FAD fungal activity [17, 37]. Similarly, total microbial respiration in our microcosms increased following the application of naphthalene on days 17 and 38 (Fig 1). The reduced response in the first 10 days of application may be due to the restructuring of microbial populations under short-term environmental stresses [39, 40]. This finding corresponds to an obvious fluctuation in the ratios of fungi to bacteria, G^+^ to G^-^ bacteria and MBC to MBN in the naphthalene microcosm (Fig 2E and 2F, Fig 3C), which is in agreement with the results showing a reduction in the radial growth of fungal cultures after 6 days of naphthalene treatment [16]. The subsequent increase in microbial respiration rates was perhaps due to the consumption of naphthalene as a carbon source by the microbial community [3, 14, 38] and the increase in fungal abundance in the naphthalene microcosm (Fig 2B, Fig 6). Moreover, the promotion effect on microbial respiration rates differed with the results Blair et al. [14] and Margesin et al. [37] owing to varying soil types (Table 3). This result suggests that naphthalene application in subalpine soil also represents an exogenous C source for soil microorganisms.

**Table 3:** Differences in the non-target effect of naphthalene additions on soil biochemical properties between in our study and six published studies.

Variables	Our study	Published studies
***Soil respiration rate***	+19.3%*	+39%* ^4^, + 12.5%* ^1^
***Microbial abundances (numbers or PLFAs)***		
Bacteria	-8.3%^ns^	+78.3%* ^1^; +10% ^ns^ ^6^
Fungi	+14.3% ^ns^	-20.1%^ns^ ^1^; -53.6* ^2^; -7% ^ns^ ^6^
FAD fungal activity	–	+123%* ^3^; +7.2%^ns^ ^4^; -62.5* ^1^;
G^+^	-10.8%^ns^	-1.3% ^ns^ ^6^
G^-^	-16.4%^ns^	+16.5% ^ns^ ^6^
***Microbial biomass***		
MBC	-1.1%^ns^	+22.7%* ^5^; +52.2* ^4^
MBN	-5.4%^ns^	none
***Enzyme activities***		
Invertase	-7.7%^ns^	none
Urease	-8.8%*	-25.6%* ^4^
Nitrate reductase	+2.2%^ns^	none
Nitrite reductase	-3.9%^ns^	none
***Carbon and nutrients***		
DOC	+1%^ns^	none
DON	-23.7%*	none
NH_4_^+^-N	-42.7%*	-78.8%* ^1^; +500%* ^3^
NO_3_^-^-N	-28.4%*	-95.2%* ^1^; +38.1%^ns^ ^3^
Inorganic N mineralization	-74.5%*	+1.4%^ns^ ^4^; -86.8%* ^1^
Total N mineralization	none	-6%* ^1^

+, positive

–, negative

*, *P* < 0.05

ns, nonsignificant.

^1^ Blair et al., 1989, flood plain forest soil

^2^ Newell et al., 1987, lake district woodlands soil

^3^ Coleman et al., 1994, corn soil

^4^ Margesin et al., 2000, arable soil

^5^ Xiong et al., 2008, shrubs and herbs soil

^6^ Cotrufo et al., 2014, grassland soil

In field experiments, naphthalene addition showed negligible direct effects on the abundance of PLFAs and carbon dynamics in field soil [3] and the abundance of total or FDA active fungi in litterbags [14]. However, the opposite results indicated that naphthalene or other biocide treatments significantly reduced the number of bacteria and FDA active fungi and the radial growth of fungi in microcosm experiments [14, 16]. Compared to the microcosm results [3, 14, 16], naphthalene application did not influence the overall microbial activities (Table 1), measured as soil microbial PLFA abundance (Fig 2) and microbial biomass (Fig 3), in our microcosm. This finding is consistent with a field experimental result [3]. Furthermore, Cotrufo et al. [3] and others [41, 42] noted that naphthalene-C might be substantially utilized by G^+^ bacteria, Actinobacteria and, to greater extent, G^-^ bacteria. Although the overall naphthalene treatment did not reach a statistically significant effect on the abundances of G^+^ and G^-^ bacteria (Table 1, Fig 6), the treatment obviously suppressed the G^+^ and G^-^ bacterial abundances before the third naphthalene application on day 38 (Fig 2C and 2D). Simultaneously, the abundances of fungi and bacteria were stimulated significantly by the naphthalene applications at the end of the experiment (Fig 2A and 2B). These disparate results can be mainly explained as follows: (1) different methods of measuring microbial abundance might have caused the different responses of microbial abundance to the naphthalene application; (2) the removal of predation by soil animals had a greater positive effect than the negative effect caused by naphthalene on the fungi [17], and thus, an overall increase was observed (Fig 6); and (3) naphthalene offered a sufficient C source to maintain fungal growth in the naphthalene microcosm [37, 38]. In addition, the response of soil MBC to the naphthalene application (Fig 3) differed from the response observed by Xiong et al. [15]; the authors found that soil MBC was obviously increased by the same naphthalene application rate (100 g m^-2^), which might be due to the higher soil organic matter content in the subalpine forest soil [19, 38] and differences in incubation times and temperatures [15].

The application of naphthalene and others biocides (e.g., triflumuron and profenofos) may introduce exogenous C and nutrients (e.g., N and P) as energy sources to microbes [15, 43] and subsequently stimulate or inhibit soil microbial activity and soil mineralization processes [44]. The naphthalene application significantly altered the soil nitrogen dynamics in our microcosms. The treatment suppressed increases in soil DON and inorganic N (NH_4_^+^-N and NO_3_^-^-N) and led to inorganic N net mineralization, which contrasted with the net immobilization result in the controls (Table 2, Fig 4B–4D). Similar results have been described by a previous study (Blair et al., 1989). The differences in DON dynamics might be due to the increase in fungal abundance following the third naphthalene application on day38, which resulted in more utilization of DON by soil microorganisms (Geisseler et al., 2010). Additionally, the decreases in extractable NH_4_^+^-N and NO_3_^-^-N during the later stage of the incubation (Fig 4C and 4D) might be attributable to the greater abundances of fungi in the treatments, which promoted microbial immobilization of inorganic N or the translocation of inorganic N from soil to microorganisms [14, 45, 46]. Moreover, soil DOC derived from dead microorganisms under naphthalene stresses might have contributed to the pulsed increase in DOC during the first 3 days of incubation in the naphthalene microcosms (Fig 4A). These results partial confirm our hypotheses that naphthalene application might have a stronger nontarget effect on the soil biochemical properties of nitrogen cycling processes than on those of carbon cycling processes in alpine forest soil. However, it should be noted that ecosystem differences and a lack of plant absorption and utilization in microcosms can result in conflicting results for nontarget effects on nitrogen cycling processes (Table 3).

Soil enzyme activity is regarded as a key indicatorof microbial activity under environmental stress [23, 47, 48]. In theory, naphthalene additions may affect extracellular enzyme activity in two ways [3]. First, reducing or removing targeted soil organism groups may directly affect other groups by altering the species-specific trophic behaviors or the interactions among these groups in the soil debris food chain [6, 49]. Second, the application of naphthalene might cause a nontarget effect on soil enzyme activity by stimulating soil respiration and microbial immobilization and abundance as well as available nutrients [3, 14]. In this study, although microbial metabolism (microbial respiration rate) and the available nutrients (organic and inorganic N) were significantly changed by the naphthalene treatments, soil invertase, nitrate reductase and nitrite reductase activities were not affected overall in the naphthalene microcosms, and similar dynamics were seen in the control (Table 1, Fig 5). Moreover, urease is an important hydrolytic biological enzyme for the conversion of organic nitrogen to available inorganic N (NH_4_^+^-N and others), whereas nitrate reductase and nitrite reductase transform NO_3_^-^-N and NO_2_^-^-N into NH_4_^+^-N [45, 50]. Studies have indicated that the production of nitrogen-degrading enzymes, such as urease, is generally controlled by the microbial assimilation of NH_4_^+^-N and NO_3_^-^-N in soil [45, 46]. Therefore, the greater fungal abundance in the naphthalene treatments (Fig 2B, Fig 6) might have stimulated the microbial assimilation of NH_4_^+^-N and NO_3_^-^-N and then significantly repress urease activity (Table 1, Fig 5). This result was accompanied by decreases in inorganic N content after the third naphthalene application on day 38 in the naphthalene microcosms (Fig 4C and 4D). In addition, although urease activity was repressed by the microbial assimilation of inorganic N, an increasing trend was observed following the incubation (Fig 5B). A likely explanation is that the microbial assimilation of inorganic N facilitated the mineralization of soil DON (Fig 4B) by urease production in the soil in the naphthalene treatment (McCarty et al., 1992). These results further demonstrate that naphthalene application might exert substantial nontarget effects on the soil biochemical properties of nitrogen cycling processes in subalpine forest soil.

## Conclusions and implications

In summary, this microcosm experiment explored the nontarget effects of naphthalene on soil microbial activities and soil nutrients by adding naphthalene to subalpine forest soil. Our results suggest that naphthalene application in subalpine soils also represents an exogenous C source for soil microbial respiration (Fig 6). The statistical analyses showed that naphthalene application did not affect the microbial activities overall, measured as soil microbial PLFA abundances and biomasses, or most enzyme activities during the whole incubation period. Overall, naphthalene application appeared to increase fungal abundance but had the opposite effect on bacterial abundance in the microcosms (Fig 6). However, the biocide application suppressed increases in DON, NH_4_^+^-N and NO_3_^-^-N contents and urease activity and led to inorganic N (NH_4_^+^-N + NO_3_^-^-N) net mineralization (Fig 6), which was contrary to the net immobilization result of the controls. Therefore, nontarget effects on soil nitrogen mineralization processes might occur when treating soil animals with naphthalene in a field experiment in subalpine forests. Caution should be taken in ascribing any changes in soil processes when using naphthalene to repel soil animals in field experiments. In addition, it should be acknowledged that there is a lack of uptake and turnover of aboveground vegetation in microcosms and whether this non-target effect exists *in situ* warrants further study. To improve the prediction of the potential nontarget effects of naphthalene application on soil biochemical properties in various ecosystems, ecosystem types, soil properties, soil-plant transformations, soil organism diversity and other factors associated with the soil biochemical cycle must be considered (Fig 6, Table 3).

## Supporting information

S1 Dataset Dataset file for the manuscript. The values represent the means and standard error (SE) of soil microcosms treated with and without naphthalene, respectively.
(XLSX)
